# Diphtheria

**Published:** 1875-03

**Authors:** J. F. Cotten

**Affiliations:** Acworth, Ga.


### DIPHTHERIA.

By J. F. COTTEN, M.D., of Acworth, Ga.

By the nomenclature of this disease is understood a peculiar inflammation of the pharynx, tonsils, uvula, fauces, nasal and eustachian passages, larynx, œsophagus, air-passages of the lungs, stomach and integuments, with an exudation of false membrane upon the mucous tissue, and, in the more malignant forms, gangrenous ulceration. We know nothing of its cause, except that it is strongly disposed to prevail as an epidemic and is perfectly independent of any condition of temperature or seasons. It is thought by some to be contagious. Such is the expressed opinion of Trousseau, Bretanneau, Guersant, Valliex, Relliet and Berthez. I think every physician who has been engaged in this recent epidemic is convinced that it is at least infectious. Such is the clear conviction of my mind. I do not believe that it is strictly contagious, like scarlet fever or small-pox, but it will spread through a family or neighborhood like influenza; and persons visiting or remaining long in houses where the disease prevails will have diphtheria. Persons spending a few days in the vicinity of the epidemic may die of the disease. Again, children surrounded by all the epidemical influences may visit other vicinities, where the disease does not appear, and entirely escape.

I do not conclude that the disease is contagious, but think it is so infectious that by constant communication with an infected person it may be contracted by the effluvia of the breath. By the observance of strict hygienic rules, caution and regularity of habit, and every prophylactic precaution, I know of some surprising immunities. Again, others have died, notwithstanding all means were used to prevent the scourge. During this epidemic one hundred or more *little darlings*, near to their mothers' hearts, have passed away.

In order to arrest its progress, every form of the healing art has been employed, from the educated and refined physician down to that form of empirical eclecticism and advertising which promises certain cures for the money. The latter class is unable to appreciate that the principal danger in diphtheria is its tendency to extend into the larynx—and success in the treatment depends upon the ability to confine it to the pharynx.

If we are called to a case before the subsidence of the febrile excitement or evidences of its spread in the larynx, we can often cure the patient, notwithstanding grave complications; but if summoned after the diphtheritic deposit is seen in the larynx, we will scarcely be able to avert a terrible and horrible death. Out of the many cases treated by myself I have lost but two. Also my friend Dr. Humphries has been equally successful. One of my fatal cases, I believe, might have been saved by tracheotomy. Had determined to operate, but could not find a canula in town. The patient literally choked to death. The other, a girl eight years old, from over-eating and exposure, relapsed after ten days' freedom from any signs of the disease, except the debility which usually follows. In this case were strong croupal symptoms, with great swelling of the tonsils, uvula, sub-maxillary and cervical glands; nasal and air-passages involved; also bloody serum constantly dripping from the nose for hours before her death.

Often the disease is preceded by fever of two or three days. Again there may be no prodromata, and the first intimation is the discovery of diphtheritic deposits on the tonsils or uvula. Had in some of my cases violent fever, delirium, hot, dry skin, full and frequent pulse. This stage of fever usually did not last long.

In none of the cases where the epidemic was most fatal did I see any eruption on the body or extremities in the later stage of the disease. Just before the thick, false membrane is detached, swelling at its height, patient gasping for breath, friends tremblingly looking for a crisis, the skin becomes spotted and purple; but this is no eruption, it is asphyxia—non-oxygenation of the blood. Just at this time the physician may pluck his patient from the grave by stripping the tonsils and uvula of the false membrane. I have detached this pseudo-membrane from its position like the cap from an acorn, excised the uvula, scarified or excised a portion of the tonsils. In such cases, if the blood has been sufficiently defibrinized to prevent a new deposit, and the larynx is not involved, the patients will usually recover very slowly.

Again, in other sections, where the disease was not so fatal, were seen papular eruption and high fever for several days beforehand. The rash in these cases looked very much like scarlet fever. Indeed, I think they were diphtheritic scarlatina. I visited a patient and found her body covered with this rash, fever for two days and a deposit appearing in the throat. In an epidemic of scarlet fever one would scarcely think of associating diphtheria with this, and yet I believe if they linger long and become debilitated the diphtheritic membrane will form wherever there is an abrasion. In the pharynx and tonsils of all these cases were thick flakes of pseudo-membrane, and also where the skin was abraded. Have seen these deposits on the lips, nose, behind the ears and in the eyes. They make a slow recovery. Believe they would not recover if not treated from the beginning with good diet, tonics, diuretics. Saw a case, first attack, violent; false membrane in the larynx, stomach, and scattered over the skin; vomited large flakes from stomach, and also a perfect cast of the trachea; no reproduction. He has voided several worms, relapsed two or three times, been anasarcous, pale and anæmic, and yet unable to walk. This is the history of many. All recover slowly, and are liable to relapse if not well guarded. The stomach and bowels are not usually deranged in the beginning; often no pain except in the throat; rarely prodromata that such a fearful disease is on hand.

During epidemics children should be daily examined, and if white or greyish spots appear, be submitted to treatment. The tendency and danger of this disease is to attack the larynx. Failure to cure often from incomplete local application. Should always attempt to confine it to pharynx; cases of pseudo-membranous croup rarely recover; diphtheria of the larynx is pseudo-membranous laryngitis. Should attempt to prevent re-formation of membrane when once dislodged. Where it is thick I have not realized much from local applications. The diseased tissue is under the membrane. If thin, remedies may be used with good effect. Am satisfied that strong caustic applications, in some instances, do more harm than good; in others we dare not omit them. In diphtheritic scarlatina strong caustics are not admissible; if persisted in will cause destructive ulceration. Have used various remedies: nit. silver in solution and stick, carbolic acid, muriatic acid in glycerine, creosote, tr. iodine, tr. ferri. chlor., bromo-chloralum comp., spirits turpentine, chlor. potassa, and many others. As a rule I prefer the solid stick of silver, mur. tr. ferri., and chlor. potassa, as local remedies. Always try to touch below the congestion if possible. Apply nitrate of silver once daily; apply every hour mur. tr. ferri., tr. myrrh, and chlor. potassa, mixed. If possible, apply to both pharynx and larynx.

Where fœtor exists use bromo-chloralum comp., or creosote and chlor. potas. in turpentine and glycerine. I use frequently chlor. potas. where the disease has spread to the stomach or the kidneys are affected. Use it in all gargles and washes; sprinkle on the throat and tonsils when possible; use it with mur. tr. ferri., and water to nasal passages; externally to the throat, flannel saturated with turpentine, kerosene oil and lard boiled with stramonium seed. Apply blisters in cases of severe swelling, but in diphtheritic scarlatina they often produce bad sores and obstinate strangury. General treatment is important and varied. Give hydrarg. chlor. mit. every two hours for several days; often combine ipecac and nit. potas; often substitute mur. ammon. for calomel; opiates not indicated; they lock up the secretions and retard the action of medicines. Have given Dover's powder at night to quiet; do not believe blood-letting indicated. The objects aimed at are to defibrinate the blood sufficiently to prevent a reproduction of the membrane. Eliminate the poison through every excretory organ; support and maintain the strength and general health.

